# Genetic parameters and principal components analysis of breeding value for birth and weaning weight in Egyptian buffalo

**DOI:** 10.5713/ajas.19.0651

**Published:** 2020-01-13

**Authors:** Mohamed Mahmoud Ibrahim Salem, Amin Mohamed Said Amin, Ayman Fouad Ashour, Mohamed Mohamed El-said Ibrahim, Mohammed Kotb Abo-Ismail

**Affiliations:** 1 Department of Animal and Fish Production, Faculty of Agriculture, University of Alexandria, Alexandria, 21545, Egypt; 2 Animal Production Research Institute, Agricultural Research Center, Dooki, Giza, 12619, Egypt; 3 Animal science Department, College of Agriculture, Food and Environmental Sciences, California Polytechnic State University, San Luis Obispo, CA 93407, USA

**Keywords:** Genetic Analysis, Growth Traits, Multivariate Techniques

## Abstract

**Objective:**

The objectives of the current study were to study the main environmental factors affecting birth weight (BW) and weaning weight (WW), estimate variance components, genetic parameters and genetic trend and to evaluate the variability and relationships among breeding value of BW and WW using principal components analysis (PCA).

**Methods:**

A total of 16,370 records were collected from 8,271 buffalo calves. Genetic parameters and breeding values were estimated using a bivariate animal model which includes direct, maternal and permanent maternal effects. These estimates were standardized and used in PCA.

**Results:**

The direct heritability estimates were 0.06 and 0.41 for BW and WW, respectively whereas direct maternal heritability values were 0.03 and 0.14, respectively. Proportions of variance due to permanent environmental effects of dam were 0.455 and 0.280 for BW and WW respectively. The genetic correlation between BW and WWs was weak approaching zero, but the maternal correlation was 0.26. The first two principal components (PC1 and PC2) were estimated utilizing the standardized breeding values according to Kaiser method. The total variance explained by the first two PCs was 71.17% in which 45.91% and 25.25% were explained by PC1 and PC2, respectively. The direct breeding values of BW were related to PC2 but those of WW and maternal breeding values of BW and WWs were associated with PC1.

**Conclusion:**

The results of genetic parameters and PCA indicate that BW and WWs were not genetically correlated and improving growth traits of Egyptian buffaloes could be achieved using WW without any adverse effect by BW.

## INTRODUCTION

Water buffalo are important source of meat, high quality milk and skin as well as other by products, not only in Egypt but also in many countries worldwide. Thus, buffalos contribute significantly in agriculture economy and human well-being in these regions. The total number of buffaloes worldwide is about 195.1 million heads, in which 97% are raised in Asia [1] and 2% (~4 million head) are reared in Egypt [1] mainly for milk and meat production. Egyptian buffaloes, known as river buffalo (*Bubalus bubalis*) contributed 39.2% and 45.2% of national total meat and milk production, respectively [1]. As 96% of buffaloes population in Egypt are owned by small holders, buffaloes play an essential role in rural economy and have challenges in establishing genetic improvement program.

Calf birth weight (BW) is one of the main breeding goals of buffalo breeders, because of its positive relationship with production and reproduction performance. Although, MMI Salem and AMS Amin [2] showed that the stillbirth incidence was similar in either light or heavy weights at birth in the Egyptian buffalo, strong genetic correlations between BW and other growth performance traits including weaning and yearling weights were reported in Pakistani Nili-Ravi buffalo [3] and Surti buffalo [4]. in addition, strong genetic correlations between BW and several production and reproduction traits such as milk yield, peak daily milk yield, lactation length, age at first calving and calving interval were reported [5]. Accurate estimations of variance components and genetic parameters such as (i.e. heritability and genetic correlation) for Egyptian buffalo growth performance traits are important to design suitable genetic improvement programs. Principal component analysis (PCA) is a multivariate data technique used to reduce the size of a set of variables by removing repeated information while maintaining the maximum variance-covariance structure of these variables [6]. The PCA has been used in the animal breeding and genetics field to reduce the size of the direct additive genetic covariance matrix in multiple trait models [6,7], and to study the relationship among predicted breeding value [8] as well as population and family structure using genomic data. Thus, the objectives of the current study were to: i) detect the main environmental factors affecting BW [7] and weaning weight (WW); ii) estimate variance components, genetic parameters and genetic trend; iii) evaluate the variability and relationships among breeding value of BW and WWs using PCA.

## MATERIALS AND METHODS

### Dataset

Data were collated from five buffalo experimental herds (El-Nattafe El-Gadid, El-Nattafe El-Kadim, Nubariya, Sids and Gimeza) belonging to the Animal Production Research Institute (APRI), Agriculture research center (ARC), Ministry of Agriculture and Land Reclamation, Egypt. A total of 16,370 records were available on 8,327 buffalo calves in which 8,271 and 8,099 records for BW and WW, respectively, were included in the analysis. These calves were born between 1980 and 2018 with pedigree having 188 sires and 2,211 dams (Table 1).

### Statistical analyses

The systematic environmental effects on growth performance traits were examined as fixed effects using least squares methods implemented in general linear model procedure of SAS [9]. These fixed effects included the effects of season of calving (4 seasons), year of calving (39 years), sex (male and female), herd (5 herds), gestation length (GL; 4 levels), weight of dam (WD; 5 levels) and parity (14 parities). The effect of GL had 4 classes; between 295 and 325 days with increment of 15 days. Whereas the WD had 5 classes between 300 and 500 kg with increment of 50 kg. The linear model was fitted as follows:

(1) $${\text{Y}}_{\text{ijklmnop}}=\mu +{\text{A}}_{\text{i}}+{\text{B}}_{\text{j}}+{\text{C}}_{\text{k}}+{\text{D}}_{\text{l}}+{\text{G}}_{\text{m}}+{\text{W}}_{\text{n}}+{\text{P}}_{\text{o}}+{\text{e}}_{\text{ijklmnop}}$$

Where, Y_ijklmnop_, the phenotypic record of BW or WW; μ, the effect of the intercept; A_i_, the fixed effect of ith season of calving; B_j_, the fixed effect of jth year of calving; C_k_, the fixed effect of kth sex; D_l_, the fixed effect of lth herd; G_m_, the fixed effect of mth gestation length; W_n_, the fixed effect of nth WD; P_o_, the fixed effect of oth parity and e_ijklmnop_, random residual assumed to be independent normally distributed with mean zero and variance σ^2^_e_. The significant fixed effects were used to form contemporary groups, which were included in genetic parameters analysis.

Variance components, heritability and breeding values were estimated using the bivariate mixed animal model using Wombat software [10]. The model can be described in the matrix notation follows:

(2) y=Xb+Za+Sm+Wc+e

And the assumptions of the variances were as:

(3) $$var\;\left[\begin{array}{c} a\\ m\\ c\\ e \end{array}\right]=\left[\begin{array}{cccc} A{\sigma^{2}}_{a} & A{\sigma^{2}}_{\mathrm{am}} & 0 & 0\\ A{\sigma^{2}}_{\mathrm{am}} & A{\sigma^{2}}_{m} & 0 & 0\\ 0 & 0 & I_{d}{\sigma^{2}}_{c} & 0\\ 0 & 0 & 0 & I_{i}{\sigma^{2}}_{e} \end{array}\right]$$

Where ***y***, a vector of BW or WW observations; ***b***, a vector of fixed effects with an incidence matrix ***X***; ***a***, a vector of random animal effects with incidence matrix ***Z***; ***m***, a vector of random dam effects with incidence matrix *S*; ***c***, a vector of random permanent environmental effects of dam with incidence matrix ***W***; and ***e***, a vector of random residual effects. ***A*** is the numerator relationship matrix between animals, ***I*** is an identity matrix, ***σ****^2^_a_* is direct additive genetic variance, ***σ****^2^_m_* is maternal additive genetic variance, ***σ****^2^_am_* is the direct × maternal genetic covariance, ***σ****^2^_c_* is maternal permanent environmental variance, and ***σ****^2^_e_* is residual variance. Direct and maternal genetic trends were estimated by regressing yearly mean estimates of breeding value of animal and dam on year of calving.

### Principal component analysis

The principal component (PC) could be used as index to evaluate animals for multiple traits [6]. First, in order to avoid the effect of traits’ different scaling and magnitudes, the estimated direct and maternal breeding values obtained for BW and WW were standardized for mean zero and unit variance. The standardization was performed according to Boligon et al [6] as follows: z_i_ = (x_i_− *χ̄*)/s_i_, where z_i_ is the standardized value of x_i_ variable, *χ̄* is the mean of ith trait, and s_i_ is the corresponding standard deviation. Then, the standardized estimated breeding values (SEBVs) were used in the PCA performed using FactoMineR package [11] in R software. Generally, the PCA reduces the information contained in estimated direct and maternal breeding values for BWs and WWs in fewer orthogonal latent variables, PCs, with minimal loss of information or variability [6]. In the current study, the PCs explained the highest percentage of variance (i.e with values greater than one eigenvalues) were used. Using the SEBVs in this analysis, each PC can generate a new value, called a principal component score, which is the result of the sum of the SEBVs of each trait weighed by their respective standardized score coefficient (SSC) [12]. Thus, the PC could be used to evaluate animals for multiple traits. The SSC of each SEBV in each principal component were obtained using the following formula:

$${SSC}_{ij}=\frac{{eigenvector}_{ij}}{\sqrt{{eigenvalue}_{j}}}$$

Where SSC_ij_ is the SSC for SEBV of *i*th trait in the *j*th PC. The principal component scores were calculated as follows:

$${\text{PCS}}_{\text{jl}}=\Sigma_{i=1}^{m}{SSC}_{ij}{SEBV}_{il}$$

Where PCS_jl_ is the principal component score for the *l*th animal in the *j*th principal component, SSC_ij_ is the SSC for EBVs of *i*th trait in the *j*th PC and SEBV_il_ is the standardised estimated breeding value of the *i*th trait for the *l*th animal.

## RESULTS AND DISCUSSION

### Environmental factors

The current study assessed the non-genetic factors affected the growth performance (i.e. BW and WW) in Egyptian buffalo (Table 2). The sex of calf significantly affected (p< 0.0001) BW and WW (p<0.05) where male calves were heavier at birth and weaning than female calves. These differences due to sex effects were in agreement with reported results by Pandya et al [4] in Surti buffalo. These differences are probably due to physiological mechanisms associated with sexual endocrine systems which cause the differences between masculinity and feminist characteristics and the natural buildup of male and female bodies [13]. Season of calving also had a significant (p<0.05) effect on both BW and WW where the heavier BW and WW buffaloes calves born in winter compared to those born in other seasons confirming the significant effect of season on buffalo calves’ growth performance reported in India [4] and Egypt [14]. Such positive effect of winter calving on BW and WW may be due to the appropriate climatic conditions especially temperature, and feeding regimes. Furthermore,, the effect of year of calving on BW had significant effect as in the results stated in other studies [4,14]. The effect of year of calving on BW reflects year to year variability with respect to feeding and management practices and climate changes. Moreover, in the current study, the effect of herd was significant (p< 0.0001) on BW but it did show significant effect on WW. The strong effect of herd may be attributed to differences in management practices among herds during the dam gestation period. Also, Gestation length had a significant effect on BW (p<0.05) but it did not have effect on WW. The calves having gestation length between 260 to 310 days had the heaviest BW, followed by those having gestation length longer than 325 days whereas the calves having gestation length below 296 days were the lightest BW. These results were in agreement with what reported in other studies on beef cattle by Jamrozik and Miller [15], where they found a positive genetic correlation between BW and GL. The current study found a significant (p<0.0001) effect of parity on BW whereas there was no effect for parity on WW. Calf BW was lower for primiparous buffaloes than those of multiparous. Furthermore, BW of calves increased with parity number. This positive relationship has reported in other studies [3,13]. The parity number and age at parturition are reproductive traits which are associated with the physiological status of the female buffaloes and positively effect on calves’ BWs. The effect of parity on BW may be due to the maturity status of the dams buffaloes as in the late parities, they have high body capacity compatible with better development of fetus [16]. In the current study we also found that, the WD had the same trend for BW, confirming the results reported in previous studies [17].

### Genetic parameters

The direct heritability estimates were 0.06 and 0.41 for BW and WW, respectively whereas direct maternal heritability values were 0.03 and 0.14, respectively (Table 3). These heritability values are in the range reported for direct heritability of BW (0.05 to 0.188) and maternal heritability (0.03 to 0.349) on other buffalo populations [4,18,19]. The low direct and maternal heritability values for BW due to maternal environmental variance being more than 7 folds of direct heritability, can be an indication that genetic selection for BW could be effective, when improving dam feeding and management practices during last phase of pregnancy might play an essential role to increase BW of calves [20]. In Brazilian buffaloes calves, Malhado et al [19] reported low heritability estimates 0.09 and 0.03 for direct and maternal heritabilities, respectively. Also, in Colombian buffalo calves, Bolívar et al [21] found low direct and maternal heritability estimates of 0.05 and 0.01 respectively. On the other hand, Pandya et al [4] and Gupta et al [18] obtained heritability values of 0.188 and 0.349, respectively, for BW of Surti Murrah buffalo. Although, the heritability estimates are population specific, the high estimates reported in Surti buffalo were probably attributed to the use of animal model without accounting for maternal effects which may overestimate the direct heritability estimates. In this study, we found a negative correlation between direct and maternal (r_am_) effects for BW (−0.16) which was reported by other studies [22]. This negative genetic correlation could be due to another negative correlation between dam and calf resulted from an adverse effect of high nutrition during early growth of calf [22].

In the current study, high (0.41) and moderate (0.14) direct and maternal heritability estimates were obtained for WW, respectively indicating that the genetic selection to improve WW is possible with good response of maternal ability [23]. Such lower estimate of maternal heritability than direct heritability indicated that the direct genetic effect has large influence on WWs in buffaloes [21]. The current heritability estimates for WW are in agreement with estimated reported in Brazilian buffalo. Nonetheless, the current estimates were higher than direct heritability estimate reported by Ashmawy and El-Bramony [24] (0.19) in Egyptian buffalo, and Pandya et al [4] (0.17) in Surti buffalo. Also, in Colombian buffalo, found direct and maternal heritability values of 0.16 and 0.04, respectively [8]. The variation in WW due to maternal environment was moderate but smaller than its value for BW, indicating that the permanent environmental influence of dam on WW has a carryover effect from birth to weaning [25]. The current results showed that the proportion of phenotypic variance due maternal permanent environmental variance (C^2^) was higher than h^2^_m_ for BW and WW, indicating that maternal environmental effect was dominant until weaning [23]. Estimates of C^2^ and h^2^_m_ for different beef breeds were close to those obtained in the current study [26]. The direct-maternal genetic correlation (r_am_) was negative for WW and higher than its value for BW. This negative r_am_ was also found in Australian beef cattle [22] and Spanish beef cattle [27]. The negative genetic correlation may be due to a negative correlation between dam and calf resulted from an adverse effect of the high nutrition during early growth of calf [22].

A low genetic correlation between BW and WW (0.05) found in the current study indicated that selection to improve WW in buffalo may not affect BW in a breeding objectives that include both traits. Such relationship is very important to avoid dystocia which may occur for calves with high BW. High estimates of genetic correlation between BW and WW of Egyptian buffalo calves were reported in other studies [18] using animal model which did not account for maternal effects. The estimates genetic correlation between growth traits in Beef cattle are usually moderate to high [3,25,27], but others were low and negative such as those by El-Saied et al [28]. The weak correlation between BW and WW may be a decrease in correlation response because of a decrease in selection intensity and population size [29]. Thus, the low genetic correlation in current study may be attributed to low selection intensity in Egyptian buffalo populations compared to beef cattle. In this study, the maternal genetic r_m_ and maternal permanent environmental r_c_ correlations were 0.262 and 0.003 respectively which were lower than estimated values 0.33 and 0.64 reported in Japanese beef cattle [25].

**Figure 1:**
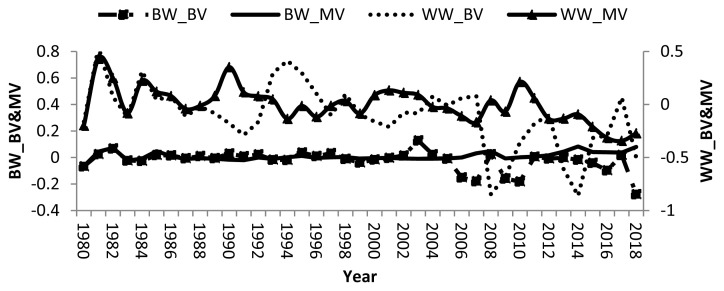
Direct and maternal genetic trends of birth and weaning weights in buffalo.

Remarkable fluctuations in breeding values of calves and dams, and negative direct and maternal genetic trend in some years were found (Figure 1). Averages of direct and maternal genetic trend were −0.003, 0.002, −0.020, and 0.001 kg/yr for BW and WW, respectively indicating that there was no specific breeding programs have been applied to improve growth traits in the studied buffalo herds. Therefore, there was no genetic improvement in either trait. Other studies showed improvement in BW and WW where the direct and maternal genetic trends for BW and WW of Brazilian buffalo were 0.006, −0.01, 0.23, and 0.05 kg/yr, respectively [19]. Nonetheless, a negative direct genetic trend was −0.03 kg/yr was reported for water buffalo in Brazil [30].

### Principal components

**Figure 2:**
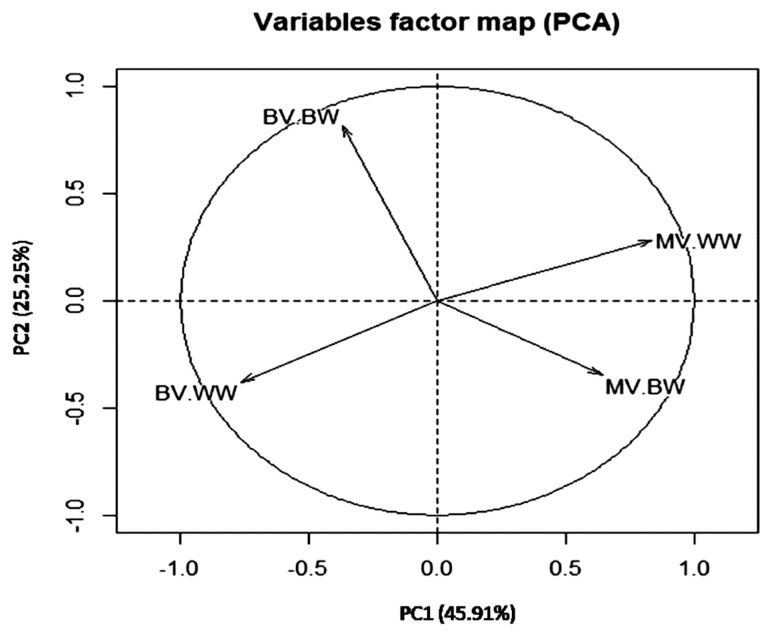
Distribution of the traits analyzed in each of the first two principal components PC1 vs PC2. BV-BW, direct breeding of birth weight; MV-BW, maternal breeding value of birth weight; BV-WW, direct breeding value of weaning weight; MV-WW, maternal breeding value of weaning weight.

The total variance explained by PCs was 71.17% in which 45.91% and 25.25% were explained by PC1 and PC2, respectively (Table 4). These results indicated that the first two PCs out of 4 showed eigenvalues greater than 1. Therefore, the first two components were sufficient to explain the most of variation in breeding value estimates for early growth traits in the current buffalo calves herds. The variation in breeding values for growth traits have been studied by Boligon et al [31] where they found the first three PCs explained 93.62% of total additive genetic variation of growth traits in Nellore cattle. In Colombian buffalo, three PCs greater explained 65.87% of the variance of breeding value for growth traits [8]. The two-dimensional graph of PC1 vs PC2 (Figure 2) and the correlation between BV, MV of both traits and PC (Table 5) showed that the direct breeding values of BW were correlated with PC2 and those for direct and maternal breeding values of BW and WW were correlated with PC1.

PC1 explained most of direct and maternal breeding value variation and was correlated with the direct and maternal breeding values of WW and maternal breeding values of BW. Thus, it could be considered as genetic index to WW and also as maternal index for BW and WW. Only 25.25% of direct and maternal breeding value variation was explained by PC2 which was correlated with direct breeding values of BW and considered as genetic index related BW. Therefore, selection to improve WW could be applied using PC1 separate from selection to improve BW in Egyptian buffalo. These results supported the reported genetic correlation between BW and WW in Table 3. The two-dimensional graph of PC1 vs PC2 illustrated in Figure 2, showed a negative correlation between maternal and genetic breeding value of BW and WW supported the direct-maternal genetic correlations reported in Table 3. A study, on Canchim cattle, reported similar trends where the first two principal components explained 73.37% of total additive genetic variance, and the PC1 was considered as genetic index for reproduction traits as well as PC2 was a genetic index correlated with body weight [12].

The PC scores for each animal in the first two principal components were calculated as:

$$\begin{array}{l} \text{PCS}1=-0.273\;{\text{BV}}_{\text{BW}\_\text{BV}}+0.472\;{\text{BV}}_{\text{BW}\_\text{MV}}-0.568\;{\text{BV}}_{\text{WW}\_\text{BV}}+0.620\;{\text{BV}}_{\text{WW}\_\text{MV}}\\ \text{PCS}2=0.811\;{\text{BV}}_{\text{BW}\_\text{BV}}-0.346\;{\text{BV}}_{\text{BW}\_\text{MV}}-0.376\;{\text{BV}}_{\text{WW}\_\text{BV}}+0.277\;{\text{BV}}_{\text{WW}\_\text{MV}} \end{array}$$

The weights of these indices were SSC for each SEBV. The larger the absolute value of the SSC, the greater the relative importance. The 2 PC scores allowing capture the main information in predicted breeding value for BW and WW. Animals with higher PCS1 could be used to improve WW, and animals with higher PCS2 could be used to improve BWs. Thus, selection with previous index could be used in genetic selection programs of Egyptian buffalo. As previously mentioned regarding the use of PCA is very practical technique to reduce the data dimensionality especially with limited number of observations in the dependent variables under the lacking of national recording system in Egypt. Also, under Egyptian breeding programs, it has been difficult to develop the economic indices and if developed they were rarely used because of the difficulty in economic weights for each trait as the Egyptian market is not stable. Thus, we can consider the PCSs as new traits which can be included in new breeding objectives and used as selection criteria under Egypt condition. Meanwhile, in other studies the PCSs can be used to reduces the genetic variance components accounting for selection bias and correlated response in correlated traits [32].

## CONCLUSION

All environmental factors affect BW, and sex, season of calving and herd affected on WW. The results indicated low heritability value for BW, high heritability value for WW and low genetic correlation between BW and WW. Moreover, 2 PCs components were sufficient to capture the breeding value variation of the studied traits. Finally PC1 and PC2 could be considered as new traits representing WW and BW, respectively. These results suggested improving growth traits of Egyptian buffaloes could be achieved using WW without any adverse effect by BW.

## Figures and Tables

**Table 1:** Description of data set for birth and weaning weights of Egyptian buffalo calves

Item	Number
Number of animals in the pedigree	9,299
Number of animals with records	8,327
Number of records	16,370
Number of sires with progeny	188
Number of sires with progeny and records	72
Mean number of progeny records per sire	115.6
Number of dams with progeny	2,211
Number of dams with progeny and records	1,373
Mean number of progeny records per dam	6.06

**Table 2:** Least square means of factors affecting birth and weaning weights of buffalo calves

Effects	Trait	

	BW	WW
Sex		
Male	36.06^a^	95.90^a^
Female	35.29^b^	95.36^b^
SEM	0.087	0.158
p-values	0.0001	0.017
Season of calving		
Autumn	35.49^b^	95.68^ab^
Spring	35.58^b^	95.45^b^
Summer	35.68^ab^	95.28^b^
Winter	35.94^a^	96.22^a^
SEM	0.126	0.228
p-values	0.025	0.035
Year of calving		
1980–1990	35.91^b^	96.14
1991–2000	34.73^d^	95.58
2001–2009	35.38^c^	95.69
2010–2018	36.75^a^	95.50
SEM	0.128	0.303
p-values	0.0001	0.721
Herd		
1	34.08^c^	95.78
2	36.29^a^	95.32
3	35.07^b^	95.06
4	34.25^c^	95.87
5	36.66^a^	96.14
SEM	0.199	0.413
p-values	0.0001	0.076
Gestation length (d)		
≤295	35.44^c^	95.57
296–310	36.01^a^	96.05
311–325	35.52^bc^	95.32
>325	35.80^ab^	95.48
SEM	0.129	0.229
p-values	0.040	0.092
Parity		
1	32.94^d^	95.15
2	34.41^c^	95.58
3	35.92^b^	95.74
4	36.13^b^	95.48
5	37.02^a^	95.82
SEM	0.140	0.265
p-values	0.0001	0.343
Dam weight		
1	30.29^e^	95.68
2	33.59^d^	95.25
3	34.91^c^	95.73
4	35.63^b^	95.28
5	36.99^a^	95.81
SEM	0.178	0.304
p-values	0.0001	0.423

SEM, standard error mean.

a–d Values within a column with different superscripts differ at p<0.05.

**Table 3:** Variance components and genetic parameters with respective standard error for birth and weaning weights in buffalo calves

Variance components	BW (Estimate±SE)	WW (Estimate±SE)
σ^2^_a_	5.01±2.26	164.9±27.24
σ^2^_m_	2.01±4.23	55.62±34.89
σ^a^_m_	−0.52±2.16	−21.74±18.64
σ^2^_c_	34.96±4.79	115.80±27.46
σ^2^_e_	35.42±1.70	87.76±15.89
σ^2^_p_	76.89±3.21	402.36±23.43
h^2^_a_	0.06±0.029	0.41±0.066
h^2^_m_	0.03±0.055	0.14±0.084
C^2^	0.45±0.053	0.28±0.068
r_am_	−0.16±0.614	−0.23±0.174
r_g_	0.06±0.217	
r_m_	0.26±0.805	
r_c_	0.003±0.127	

BW, birth weights; WW, weaning weights; SE, standard error; σ^2^_a_, direct genetic variance; σ^2^_m_, maternal genetic variance; σ_am_, direct × maternal genetic covariance; σ^2^_c_, maternal permanent environmental variance; σ^2^_e_, residual variance; σ^2^_p_, phenotypic variance; h^2^_a_, direct heritability; h^2^_m_, maternal heritability; C^2^, proportion of phenotypic variance due maternal permanent environmental variance; h^2^_t_, total heritability; r_am_, genetic correlation between direct × maternal effect; r_g_, direct genetic correlation between birth and weaning weights; r_m_, maternal genetic correlation between birth and weaning weights; r_c_, maternal permanent environmental correlation between birth and weaning weights.

**Table 4:** Eigenvalues and variance proportions for the principal components of the standardized breeding values

Principal components	Eigenvalue	Variance (%)	Cumulative variance (%)
1	1.836	45.91	45.91
2	1.01	25.25	71.17
3	0.743	18.59	89.76
4	0.409	10.23	100

**Table 5:** Correlation coefficients between standardized breeding values of the studied traits with the first 2 principal components

Trait	PC1	PC2
BW_BV	−0.370	0.82
BW_MV	0.64	−0.35
WW_BV	−0.77	−0.38
WW_MV	0.84	0.28

PC, principal component; BW_BV, direct breeding of birth weight; BW_MV, maternal breeding value of birth weight; WW_BV, direct breeding value of weaning weight; WW_MV, maternal breeding value of weaning weight.
